# Capnography activation is improved by better ventilator interface ergonomics

**DOI:** 10.1186/cc9559

**Published:** 2011-03-11

**Authors:** E Hodge, M Blunt, P Young

**Affiliations:** 1 Queen Elizabeth Hospital, King's Lynn, UK

## Introduction

In critical care, capnography is recommended [1]. Upon intubation this is important to rapidly confirm endotracheal tube position. Often capnography is built into critical care ventilators, but as these are frequently used for non-invasive ventilation it is necessary that this monitoring may be switched off and on. We postulated that the ease with which this could be done would relate to the ergonomic design of the ventilation interface and compared the Drager Evita 4 and Drager V500. The Evita 4 has a button hidden within the alarm limits section, whereas on the V500, which has locally configurable interface, this had been placed on the main screen.

## Methods

Thirty-one nursing and medical ICU staff were studied. The ventilator was set up in a controlled mode with the default front screen visible with capnography disabled. The time to successful activation of capnography was recorded. Each subject performed the same test on both ventilators in a randomized crossover design.

## Results

**Figure 1:**
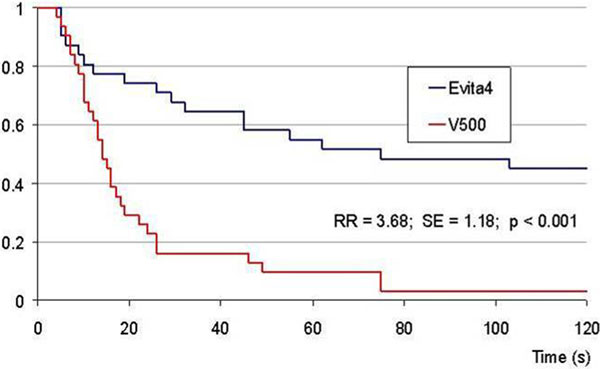
**Survival analysis for time to capnography activation for Evita 4 and V500**.

More subjects failed to activate capnography within 120 seconds with the Evita 4 compared with the V500 (14 vs. 1) and survival analysis identified significantly faster time to successful activation in the V500 (see Figure 1). Analysis identified no period effect due to the crossover design.

## Conclusions

Despite the extensive experience and training on the Evita 4, many subjects were not able to activate capnography within 2 minutes; however, by configuring the screen of the V500 this was almost eliminated in staff even without specific training. Immediate availability of capnography is an important safety issue and manufacturers should consider this in the ergonomic design of their equipment interfaces.
